# High-throughput transcriptomic and proteomic profiling of mesenchymal-amoeboid transition in 3D collagen

**DOI:** 10.1038/s41597-020-0499-2

**Published:** 2020-05-27

**Authors:** Vladimír Čermák, Aneta Gandalovičová, Ladislav Merta, Karel Harant, Daniel Rösel, Jan Brábek

**Affiliations:** 1 Department of Cell Biology, Charles University, Viničná 7, Prague, Czech Republic; 2 Biotechnology and Biomedicine Centre of the Academy of Sciences and Charles University (BIOCEV), Průmyslová 595, 25242 Vestec u Prahy, Czech Republic; 3 Proteomics Core Facility, Faculty of Science, Charles University, Prague, Czech Republic

**Keywords:** Metastasis, RNA sequencing, Proteomics, Mass spectrometry, Cell migration

## Abstract

The plasticity of cancer cell invasion represents substantial hindrance for effective anti-metastatic therapy. To better understand the cancer cells’ plasticity, we performed complex transcriptomic and proteomic profiling of HT1080 fibrosarcoma cells undergoing mesenchymal-amoeboid transition (MAT). As amoeboid migratory phenotype can fully manifest only in 3D conditions, all experiments were performed with 3D collagen-based cultures. Two previously described approaches to induce MAT were used: doxycycline-inducible constitutively active RhoA expression and dasatinib treatment. RNA sequencing was performed with ribo-depleted total RNA. Protein samples were analysed with tandem mass tag (TMT)-based mass spectrometry. The data provide unprecedented insight into transcriptome and proteome changes accompanying MAT in true 3D conditions.

## Abstract (summary)

**Table:** 

Measurement(s)	gene-expression profile endpoint • protein expression profiling • Proteome • transcriptome
Technology Type(s)	RNA sequencing • MSn spectrum • mass spectrometry
Factor Type(s)	doxycycline-inducible expression of EGFP-RhoA G14V gene • dasatinib treatment
Sample Characteristic - Organism	Homo sapiens

Machine-accessible metadata file describing the reported data: 10.6084/m9.figshare.12084927

## Background & Summary

Cancer is the result of deregulation of cellular processes, namely of cell proliferation, differentiation, survival/apoptosis, metabolism and migration^1^. Aberrant invasive behaviour of cancer cells can result in metastasis, a process responsible for tumour dissemination and related mortality, accounting for approx. 90% deaths from cancer. Using ancient, evolutionary conserved mechanisms, cancer cells invade the extracellular matrix (ECM) either as cell clusters or sheets, described as collective invasion, or alternatively, migrate as individual cells^2^. When migrating individually, cells can adopt either the protease-dependent mesenchymal mode or the protease-independent amoeboid mode. In general, mesenchymally invading cells display a fibroblast-like morphology with a distinct leading and trailing edge^3^. They form actin-rich protrusions that engage in stable cell-ECM contacts mediated mostly by integrins^4^. Mesenchymal cells further form invasive structures, such as invadopodia and podosomes, that produce proteolytically active enzymes, most commonly matrix metalloproteinases^5,6^. The secretion of such enzymes serves to digest the surrounding ECM and form tracks large enough for cell body translocation^7^.

Unlike mesenchymal invasion, amoeboid invasion does not fully depend on proteolytic digestion and formation of stable cell-ECM adhesions. The cells rather take advantage of pre-existing pores in the ECM and dynamically change their cell body to squeeze through^8,9^. Amoeboid cells may display enhanced actomyosin contractility due to persistent activation of the RhoA/ROCK pathway, leading to increased hydrostatic pressure that drives formation of membrane blebs^10,11^. However, a few different subtypes of the amoeboid migratory phenotype have been described and diverse theories explaining the physical mechanism of cell translocation in amoeboid cells have been suggested^12^. So far, no specific biochemical marker of the phenotype has been shown to be a universal feature of amoeboid cells arising from different cell types. Importantly, cancer cell invasion is responsive to surrounding conditions and transitions between the individual modes can occur. The mesenchymal-amoeboid (MAT) or amoeboid-mesenchymal (AMT) transitions can be induced by modulating the activity of key signalling hubs, such as the Rho GTPases, or by targeting necessary mechanisms of either invasion mode^13,14^. The plasticity of invasion is presumably the main reason why clinically usable anti-metastatic treatment strategies are still unavailable^15^. Despite the large effort to reveal signalling underlying invasive behaviour of cells, understanding of cancer cell invasion plasticity is still insufficient, mainly due to the scarcity of results obtained from more *in vivo*-like 3D cell culture conditions. To date, there are only three published works reporting gene expression profiling of amoeboid cells^16–18^. While these data provided the first insight into the transcriptome of amoeboid cells, they were not obtained from three-dimensional (3D) cultures, an essential requirement to get the most relevant results.

**Fig. 1:**
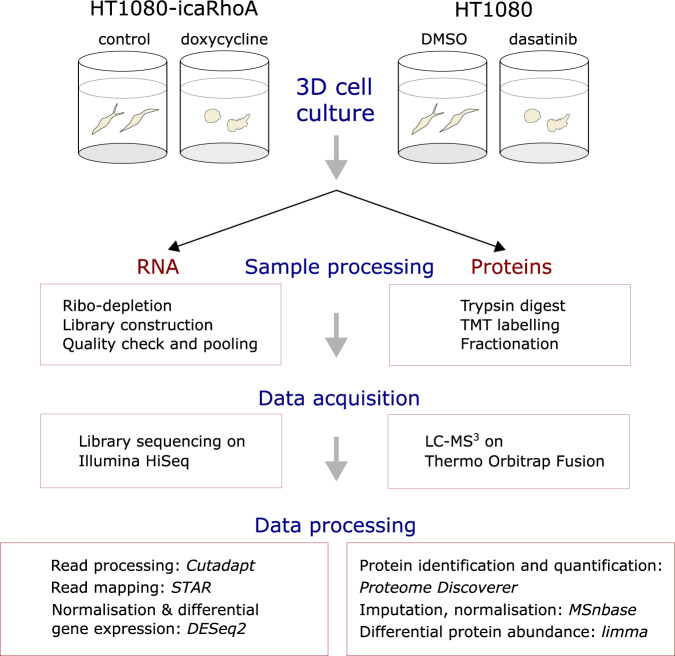
Schematic overview and experimental design of the study.

To gain more insight into molecular level adaptation of cancer cells to the amoeboid state, we performed large scale transcriptomic and proteomic profiling of HT1080 fibrosarcoma cells after MAT in 3D cell culture (Fig. 1). In order to discern treatment-specific effects, we used two experimental treatments that are sufficiently effective in inducing MAT and compatible with cell viability in 3D collagen gels. The first treatment was doxycycline-inducible constitutively active RhoA (icaRhoA) gene expression; RhoA-ROCK pathway is known to play a key role in amoeboid migration^13,19^ and constitutively active RhoA expression has been shown to induce amoeboid morphology in glioblastoma cells and effective MAT in HT1080^3,20^. The second, very different treatment was that with dasatinib, a Src kinase inhibitor, that has been previously also shown to induce MAT^21,22^. The cells were kept for 48 hours in 3D collagen without or with the MAT-inducing treatment and then the whole samples including the collagen and any extracellular material were homogenized and further processed for RNA sequencing or mass spectrometry analysis. Total RNA was depleted of rRNA, converted into a stranded cDNA library and sequenced with Illumina HiSeq sequencer. Protein lysates were trypsin-digested, TMT-labelled, fractionated and analysed on Thermo Orbitrap Fusion mass spectrometer.

Overall, our work provides data for an unprecedented comparison of parallel mesenchymal and amoeboid transcriptomes and proteomes obtained from 3D conditions to reveal new details of the transition regulation, the impact of the transition on biological properties of the cells in terms of gene expression and protein abundance, and possibly discover potential new therapeutic opportunities.

## Methods

### Cells, DNA constructs and transfection

HT1080 cells were maintained in DMEM (4.5 g/l glucose, pyruvate) supplemented with 10% fetal bovine serum and 50 μg/ml gentamicin (all from Sigma) at 37 °C in humidified atmosphere with 5% CO_2_. The cultures were regularly tested for mycoplasma contamination. 3D cell culture experiments were performed with rat tail collagen (SERVA) at concentration 1 mg/ml and DMEM supplemented with 1% fetal bovine serum, 15 mM HEPES and 50 μg/ml gentamicin. Stably transfected cells were prepared by lentiviral transduction using the second-generation packaging system (pLVX constructs, Tet-On Advanced Gene expression system, Clontech). The DNA transfections were performed with polyethylenimine (Polysciences, Inc.). All populations of stably transfected cells were further enriched for the respective encoded fluorescence with a cell sorter. Cells bearing inducible constructs were transiently induced with doxycycline before the sorting. In all experiments, cells stably transfected with the doxycycline-inducible EGFP-RhoA G14V fusion construct were treated with 250 ng/ml doxycycline (Sigma). In dasatinib experiments, HT1080 cells stably expressing LifeAct-mCherry exogene^23^ (further referenced just as HT1080) were treated with 1 μM dasatinib (LC Laboratories) in DMSO or equivalent volume of DMSO only (0.1% final concentration). All plasmids used in the study were constructed in the lab with standard molecular cloning procedures; details as well as the plasmids themselves are available upon request.

### Time-lapse microscopy in 3D collagen matrix

To record cell invasion in 3D collagen, control and doxycycline- and dasatinib-treated cells with or without protease inhibitor GM6001 (10 µM) were imaged every 5 minutes using JuLI FL, an in-incubator microscope (NanoEnTek Inc.). For digital holographic microscopy, cells were embedded in collagen as stated above and left to adjust to 3D conditions for at least 12 hours. Images were acquired automatically in 80–90 second intervals. Cells were imaged using QPHASE (TESCAN Brno, s.r.o.) a multimodal holographic microscope based on CCHM technology as described previously^3^.

### Cell morphology in 3D collagen matrix

100,000 cells were seeded in 250 μl of collagen matrix in a 48-well plate. Hoffman modulation contrast microscopy images were taken 48 hours later from approx. 20 planes along z-axis. Cell morphology was assessed by measuring the ratio of the maximum length and maximum width manually using Fiji software^24^. At least 300 cells were analysed for each condition.

### RNA extraction and sequencing

One million HT1080 cells were cultured in a 500 μl 3D collagen gel for 48 hours in a 24-well plate. Gels from two wells were transferred into one 2 ml tube and homogenized with Tissue Tearor (BioSpec Products) in 600 μl of RNA extraction solution (60% v/v water-saturated phenol, 3.25 M guanidine thiocyanate, 400 mM sodium acetate buffer pH 4.0, 0.4% w/v N-lauroylsarcosine, 160 mM 2-mercaptoethanol) plus 100 μl of 6.1 M sodium chloride. 200 μl of chlorophorm was added to approx. 1 ml of the lysate and the mixture was vortexed vigorously for 10 seconds. After 30-minute centrifugation at 18,000 g at 4 °C, the polar upper phase was transferred to a new tube, volume adjusted to 800 μl with RNase-free water, RNA was precipitated with 600 μl of isopropanol and recovered by centrifugation at 18,000 g for 30 min at 4 °C. The RNA pellet was washed three times with 600 μl of 75% ethanol and air-dried. Next, the RNA was treated with DNAse I to remove any possible genomic DNA contamination. To this end, the pellet was directly dissolved in 100 μl of solution containing 4 units of DNAse I (Thermo Fisher Scientific) in manufacturer-provided reaction buffer and incubated at 37 °C for 30 min. After that, the RNA was re-purified with RNeasy Protect Mini Kit (Qiagen) according to the manufacturer’s instructions and eluted in 50 μl of RNase-free water. Stranded, Illumina HiSeq-compatible library was constructed with ScriptSeq Complete (Human/Mouse/Rat) library preparation kit (Epicentre) according to the manufacturer’s instructions. The quality and size distribution of sequencing libraries was analysed with Agilent BioAnalyzer 2100. An equimolar pool of 6 sample libraries was sequenced on one whole lane of Illumina HiSeq 2000/2500 series sequencer in high output, paired mode (2 × 100 cycles in case of the inducible caRhoA cells and 2 × 125 cycles in case of the DMSO/dasatinib treated cells). Raw reads were trimmed of adapter sequences with Cutadapt^25^ (version 1.15), quality-checked with fastqc and mapped to human genome version GRCh38.91 with the STAR short read aligner^26^ version 2.5.4b with default settings and output extended with read counts per gene. Complete adapter-trimmed fastq data are available from the ArrayExpress database at EMBL-EBI under accession number E-MTAB-6823^27^. The samples and related files are summarized in Table 1.

**Table 1:** Summary of RNA sequencing data.

Sample	Cells	Treatment	Protocols	ENA	BioSD
1	HT1080-icaRhoA	none	P-MTAB-75206 to P-MTAB-75211	ERS2515967	SAMEA4695791
2	HT1080-icaRhoA	doxycycline	P-MTAB-75206 to P-MTAB-75211	ERS2515964	SAMEA4695788
3	HT1080-icaRhoA	none	P-MTAB-75206 to P-MTAB-75211	ERS2515968	SAMEA4695792
4	HT1080-icaRhoA	doxycycline	P-MTAB-75206 to P-MTAB-75211	ERS2515966	SAMEA4695790
5	HT1080-icaRhoA	none	P-MTAB-75206 to P-MTAB-75211	ERS2515969	SAMEA4695793
6	HT1080-icaRhoA	doxycycline	P-MTAB-75206 to P-MTAB-75211	ERS2515965	SAMEA4695789
7	HT1080-LifeAct-mCherry	DMSO	P-MTAB-75206 to P-MTAB-75211	ERS2515962	SAMEA4695786
8	HT1080-LifeAct-mCherry	dasatinib	P-MTAB-75206 to P-MTAB-75211	ERS2515960	SAMEA4695784
9	HT1080-LifeAct-mCherry	DMSO	P-MTAB-75206 to P-MTAB-75211	ERS2515963	SAMEA4695787
10	HT1080-LifeAct-mCherry	dasatinib	P-MTAB-75206 to P-MTAB-75211	ERS2515958	SAMEA4695782
11	HT1080-LifeAct-mCherry	DMSO	P-MTAB-75206 to P-MTAB-75211	ERS2515961	SAMEA4695785
12	HT1080-LifeAct-mCherry	dasatinib	P-MTAB-75206 to P-MTAB-75211	ERS2515959	SAMEA4695783

### High-throughput proteomic profiling

One million HT1080 cells were cultured in a 500 μl 3D collagen gel for 48 hours in a 24-well plate. Gels from two wells were transferred into one 2 ml tube, mixed with equal volume of 2x TEAB buffer (200 mM triethylammonium bicarbonate pH 8.5, 4% sodium deoxycholate, both from Sigma) and homogenized with Tissue Tearor (BioSpec Products). After 15-minute centrifugation at 18,000 g at 4 °C, supernatant was transferred into a new tube and kept frozen at −80 °C before further processing. Protein samples were trypsin-digested, TMT-labeled, fractionated and analysed on Orbitrap Fusion mass spectrometer (Thermo Fisher Scientific) as described previously^28–30^. Specifically, sample volume containing 100 μg of proteins was precipitated by four volumes of cold acetone (overnight at −20 °C). After centrifugation the pellets were washed with 80% acetone and let dry. Samples were resuspended in 100 mM TEAB (Triethylammonium bicarbonate, Thermo #90114) reduced with 5 mM TCEP (Tris(2-carboxyethyl)phosphine hydrochloride, Sigma #4706) for 30 min at 60 °C and alkylated with 10 mM MMTS (S-Methyl methanethiosulfonate, Sigma #64306) for 10 min at room temperature. Proteins were digested with trypsin (trypsin:protein ratio 1:50) overnight at 37 °C. TMT label was added according to manufacturer protocol. After 60 min, the labelling reaction was stopped by addition of hydroxylamine. All samples were pooled and vacuum dried. Samples were desalted on Michrom C18 Opti Trap Macro (Optimize Technologies 10-04818-TN). Peptides were fractionated as follows. 100 μl volume of a sample containing approx. 250 μg of peptides was injected onto a C18 column (kinetex 1.7 μm, EVOC18, 150 × 2.1 mm) and separated with linear gradient from 0% A (20 mM ammonium formate, 2% acetonitrile pH 10) to 50% B (20 mM ammonium formate, 80% acetonitrile pH 10) in 32 min with flow rate 300 μl/min 32 fractions were collected and pooled into 8 fractions. Resulting fractions were dried and resuspended in 20 μl of 1% TFA. Nano reversed phase column (EASY-Spray column, 50 cm × 75 μm ID, PepMap C18, 2 μm particles, 100 Å pore size) was used for LC/MS analysis. Mobile phase buffer A was composed of water, 2% acetonitrile and 0.1% formic acid. Mobile phase buffer B was composed of 80% acetonitrile in water and 0.1% formic acid. Samples were loaded onto the trap column (AcclaimPepMap300, C18, 5 μm, 300 Å wide pore, 300 μm × 5 mm) at a flow rate of 15 μl/min. Loading buffer was composed of water, 2% acetonitrile and 0.1% trifluoroacetic acid. Peptides were eluted with gradient of B from 2% to 60% over 240 min at a flow rate of 300 nl/min. Eluting peptide cations were converted to gas-phase ions by electrospray ionization and analyzed on a Thermo Orbitrap Fusion (Q-OT-qIT, Thermo) mass spectrometer. Spectra were acquired with 4 seconds duty cycle. Full MS spectra were acquired in orbitrap within mass range 350–1,400 m/z with resolution 120,000 at 200 m/z and maximum injection time 50 ms. Most intense precursors were isolated by quadrupole with 1.6 m/z isolation window and fragmented using CID with collision energy set to 30%. Fragment ions were detected in ion trap with scan range mode set to normal and scan rate set to rapid with maximum injection time 50 ms. Fragmented precursors were excluded from fragmentation for 60 seconds. For quantification information of a TMT label 10 most intense fragments were isolated (simultaneous precursor selection) and fragmented in HCD on 65% energy, maximum accumulation time 140 ms, and fragments were measured in orbitrap on 60 K resolution. Raw data were processed in Proteome Discoverer 2.1. TMT reporter ions ratios were used for estimation of relative amount of each protein. Searches were done with the Human Uniprot reference database and a common contaminant database. Modification were set: peptide N terminus, lysine (unimod #737) and cystein (unimod #39) as static, and methionine oxidation (unimod #1384) and protein N-terminus acetylation (unimod #1) as variable. Raw data are available from the PRIDE database^31^ under identifier PXD010425^32^. The samples and related files are summarized in Table 2.

**Table 2:** Summary of mass spectrometry proteomic data.

Sample	Cells	Treatment	PRIDE RAW file
R1	HT1080-icaRhoA	none	D1_170107152223
R2	HT1080-icaRhoA	doxycycline	D2_170107195536
R3	HT1080-icaRhoA	none	D3_170108002847
R4	HT1080-icaRhoA	doxycycline	D4_170107104914
R5	HT1080-icaRhoA	none	D5_170108050200
R6	HT1080-icaRhoA	doxycycline	D6_170108093514
D1	HT1080-LifeAct-mCherry	DMSO	R1_170110145546
D2	HT1080-LifeAct-mCherry	dasatinib	R2_170110192857
D3	HT1080-LifeAct-mCherry	DMSO	R3_170111000208
D4	HT1080-LifeAct-mCherry	dasatinib	R4_170111043519
D5	HT1080-LifeAct-mCherry	DMSO	R5_170111090830
D6	HT1080-LifeAct-mCherry	dasatinib	R6_170111134141

### Immunoblotting

One million cells were cultured in a 500 μl 3D collagen gel for 48 hours in a 24-well plate. Gels from two wells were transferred to tubes containing 500 μl of 2x SDS lysis buffer (2% SDS, 20% glycerol, 120 mM Tris, pH = 6.8) and homogenized using Tissue Tearor (BioSpec Products). After 10-minute centrifugation (18,000 rcf, 10 °C) 900 ul of the solution was transferred to a fresh tube and protein concentration in the lysate was determined using the DCTM Protein Assay (Bio-Rad Laboratories). The lysates in each series were adjusted to the same protein concentration with 1x SDS lysis buffer. DTT (final concentration 50 mM) and bromophenol blue (final concentration 30 μM) were added, and the samples were incubated at 95 °C for 10 min. Samples were separated on 10% or 12% SDS-polyacrylamide gels and transferred onto nitrocellulose membrane. Non-specific binding was blocked by incubation of the membranes for 60 min at room temperature in Tris-buffered saline (TBS) containing 4% BSA or 5% non-fat dry milk. The membranes were incubated with a primary antibody in 4 °C overnight, washed three times in Tris-buffered saline with Tween-20 (TBST) and incubated for 75 min with HRP-conjugated secondary antibody at room temperature. Membranes were washed with TBST two times, with TBS one time and developed using Amersham^TM^ Imager 600 (GE Healthcare) and SuperSignal^TM^ Femto Maximum Sensitivity Substrate (Thermo Fisher Scientific) or Western Bright TMECL (Advansta) HRP substrates. For probing the total protein level after a phosphoprotein detection and for loading control (GAPDH) detection, membranes were stripped in stripping buffer (200 mM NaOH) at 42 °C for 10 min. The primary antibodies used were as follows: GAPDH (Thermo Fisher Scientific MA5-15738), Caspase-3 (Cell Signaling Technology #9662), Fra-1 (Developmental Studies Hybridoma Bank PCRP-FOSL1-1E3), C/EBPβ (Abcam Ab32358), RhoA (Cell Signaling Technology #2117), GDF15 (Thermo Fisher Scientific PA5-17065) and MX1 (Cell Signaling Technology #37849). Quantification of band signals was performed using Multi Gauge software (Fujifilm, Tokyo, Japan). Band intensities of specific proteins were normalized to the GAPDH protein signal. Log-transformed signal values from at least three experiments were analyzed with paired Student’s t-test. The results were presented as geometric means of fold change values with respect to control samples and the uncertainty was expressed as geometric standard errors.

### Activated RhoA detection

One million cells were cultured in a 500 μl 3D collagen gel for 48 hours in a 24-well plate. Gels from two wells were transferred to tubes containing 500 μl of 2x Triton-X100 lysis buffer (2% Triton X-100, 100 mM Tris, 300 mM NaCl, pH = 7.1, protease inhibitors) and homogenized using Tissue Tearor (BioSpec Products) on ice. After 10-minute centrifugation (18,000 g, 10 °C) 800 μl of the solution was transferred to a fresh tube, protein concentration in the lysate was determined using the DCTM Protein Assay (Bio-Rad Laboratories) and adjusted to the same value in each series with 1x Triton X-100 lysis buffer. 50 μl of the lysate was transferred to a fresh tube (total lysate control) and the rest was incubated with rhotekin-bound GST-beads at 4 °C for 45 min. Beads were separated by brief centrifugation and washed two times with 1x Triton X-100 lysis buffer. Finally, beads were resuspended in 1x Laemmli sample buffer (0.35 M Tris-HCl, pH = 6.8, 10% SDS, 40% glycerol, 0.012% bromophenol blue) with DTT (50 mM) and incubated at 95 °C for 10 minutes. Samples were further processed according to the immunoblotting protocol described above.

### Statistical analysis

To estimate differential gene expression from RNA sequencing data a workflow based on the STAR aligner and DESeq2 R package was used as described previously^18^. Mass spectrometry data pre-processed with Proteome Discoverer 2.1 were imported into R environment, imputed and normalized with the MSnbase package^33^. Differences in protein levels were estimated with moderated t-test statistics using the limma package^34^.

## Data Records

Time-lapse movies documenting migratory phenotypes of the cells, uncropped immunoblot images, other supporting data referenced in the text, and complete results of differential transcript and protein level analyses were deposited in the Figshare^35^ repository. Adapter-trimmed RNA sequencing data have been deposited in the ArrayExpress database at EMBL-EBI^27^. Raw proteomic data have been deposited in the PRIDE database via the ProteomeXchange Consortium^32^. Besides the described data, the PRIDE record contains also a series of datasets obtained from HT1080-icaRhoA cells cultured on 2D collagen (three pairs of doxycycline-untreated/treated cells).

## Technical Validation

**Fig. 2:**
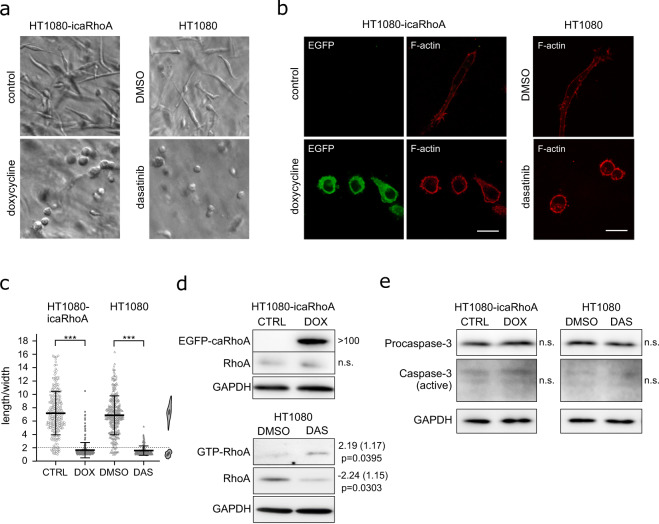
Characterization of HT1080 cells induced to undergo MAT. (**a**) Representative wide-field images of cells in 3D collagen without or with induction of MAT. (**b**) Confocal microscopy of cells stained with fluorescently labelled phalloidin. (**c)** Quantification of cell morphology in 3D collagen (Student’s t-test, p-values: ***p < 0.001, **p < 0.01, *p < 0.05). (**d**) Immunoblotting detection of RhoA protein in cell lysates. In cells treated with DMSO or dasatinib, the active, GTP-bound RhoA was separated using GST-Rhotekin 1 RBD pulldown. Numbers next to blots indicate average fold change, standard error (both geometric) and p-value of paired t-test. (**e**) Immunoblotting detection of Caspase-3 in cell lysates. All results come from 48-hour experiments. Scale bar 20 μm in all cases. Abbreviations: CTRL – control, DOX – doxycycline, DAS – dasatinib, n.s. – not significant.

**Fig. 3:**
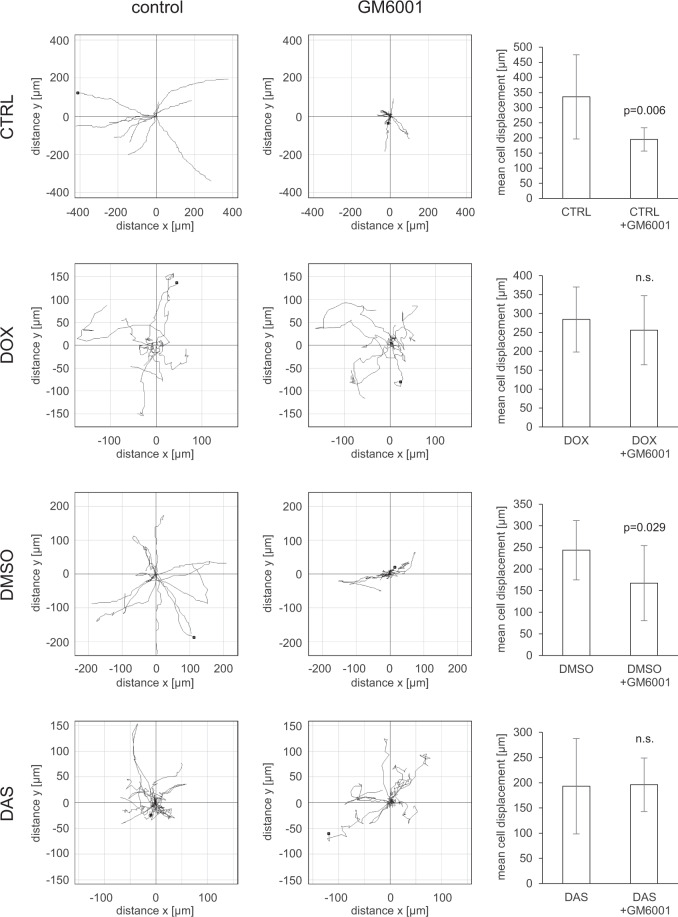
Quantitative analysis of cell migration. HT1080-icaRhoA cells and HT1080 cells were embedded in rat-tail collagen (1 mg/ml) with or without GM6001. 12 hours later, migration of cells of the mesenchymal phenotype (HT1080-icaRhoA CTRL, HT1080 DMSO) and amoeboid phenotype (HT1080-icaRhoA DOX, HT1080 DAS) was monitored by wide-field microscopy for 15 hours. Track plots were generated using Chemotaxis Tool in ImageJ. Differences in the mean length of the tracks were analysed with two-tailed Student’s t-test, α = 0.05, n = 9–13. Error bars – standard deviation. Representative results of three independent experiments.

Induction of icaRhoA in otherwise mesenchymally migrating HT1080 fibrosarcoma induced effective MAT, i.e. cell rounding, membrane blebbing and amoeboid migration resistant to extracellular protease inhibitor (GM6001) in 3D collagen in the vast majority of the cell population (Figs. 2a–c, 3 and additional files available from Figshare^35^: time-lapse Movies 1 and 2). Treatment of HT1080 cells with 1 µM dasatinib produced very similar effects (Figs. 2a–c, 3 and time-lapse Movies 1 and 3 plus the files “Src activity in MAT-induced cells” all available from Figshare^35^). We also tested the effect of other Src inhibitors and found that they were much weaker MAT inducers than dasatinib (see the file “Src inhibitors comparison” available from Figshare^35^). The different potential of the Src inhibitors to induce MAT could be attributed to their different effect on Src structure and localization^36^. The induction of the EGFP-RhoA G14V fusion protein in HT1080-icaRhoA cells was verified with immunoblotting (Fig. 2d). Activation of RhoA – an expected feature of the amoeboid phenotype – in dasatinib-treated cells was confirmed with GST-Rhotekin 1 Rho binding domain (RBD)-based pulldown assay (Fig. 2d, see also the file “RhoA activity in MAT-induced cells” available from Figshare^35^). To verify that the observed membrane blebbing is not due to initiation of apoptosis, we detected Caspase-3 in protein lysates from the cells in 3D collagen. While we easily detected the non-cleaved pro-form in all samples, only a faint signal of the active, cleaved form of Caspase-3 could be detected after a very long blot exposure in all samples with no significant differences (Fig. 2e).

**Fig. 4:**
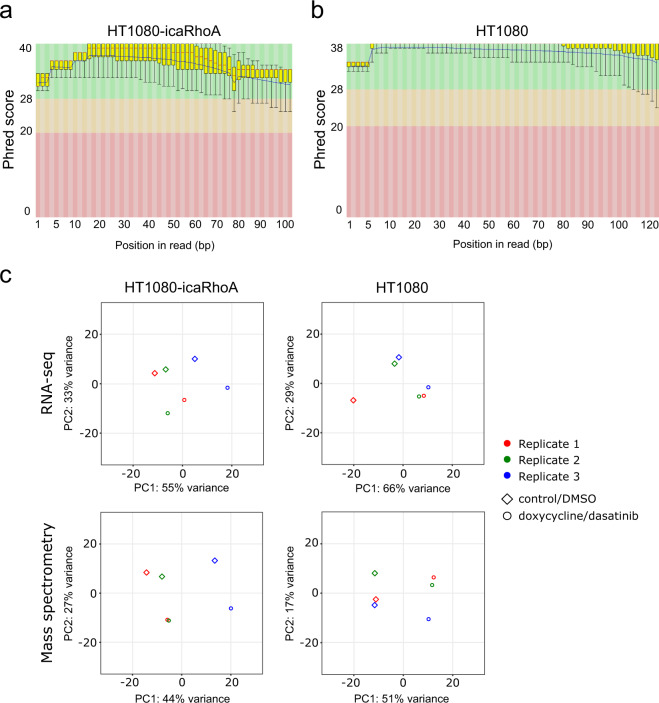
Transcriptomic and proteomic profiling of HT1080 cells of mesenchymal and amoeboid migratory phenotype. (**a**) Per base sequence quality of RNA sequencing reads expressed as Phred score by position, HT1080-icaRhoA control sample 1, first reads. (**b**) Same as (**a**), HT1080 DMSO control sample 1. (**c**) Principal component analysis of gene expression and protein abundance profiles. The normalized expression abundance of mRNAs and proteins was used. Point shapes indicate treatment; point colours mark individual pairs of biological replicates.

RNA sequencing of control and MAT-induced samples (three pairs of independent biological replicates for each treatment) yielded 31–46 million paired-end reads per sample. Raw reads trimmed of adapter sequences were quality-checked with FastQC software^37^. FastQC’s plots of Phred scores (the standard measure of position reading confidence) by position (Fig. 4a,b) showed typical high-quality profiles with decreasing quality towards the ends of reads. Note that the inducible caRhoA series samples were sequenced with 2 × 100 cycles while the dasatinib treatment series samples were sequenced later with improved version of the sequencing chemistry enabling longer read length (2 × 125 cycles). The mapping metrics of STAR aligner showed an average paired read length of 191 bases for inducible caRhoA samples and 235 bases for dasatinib treatment series samples. In average, the aligner uniquely mapped 90.7% of the fragments to human genome, 5.3% fragments were multi-mapped, and 3.7% fragments were excluded as too short. The complete mapping metrics is listed in Table 3.

**Table 3:** RNA sequencing raw data and mapping metrics.

Sample	Cells	Treatment	Reads pairs total	Average read input length	% mapped	% multi-mapped	% too short
1	HT1080-icaRhoA	none	36,639,825	192	89.90	5.40	4.20
2	HT1080-icaRhoA	doxycycline	36,165,871	189	90.55	4.92	3.88
3	HT1080-icaRhoA	none	30,919,393	189	90.62	4.95	3.85
4	HT1080-icaRhoA	doxycycline	36,664,126	193	88.91	5.75	4.93
5	HT1080-icaRhoA	none	36,506,595	189	89.58	3.89	6.23
6	HT1080-icaRhoA	doxycycline	37,336,035	191	92.32	3.18	4.20
7	HT1080-LifeAct-mCherry	DMSO	39,167,799	245	95.18	2.83	1.85
8	HT1080-LifeAct-mCherry	dasatinib	35,644,171	235	90.21	6.67	2.84
9	HT1080-LifeAct-mCherry	DMSO	41,488,195	232	90.54	6.32	2.88
10	HT1080-LifeAct-mCherry	dasatinib	42,412,264	235	91.89	4.98	2.92
11	HT1080-LifeAct-mCherry	DMSO	42,742,403	232	88.94	7.72	3.02
12	HT1080-LifeAct-mCherry	dasatinib	45,846,680	231	89.71	6.87	3.08

Mass spectrometry sample quality was checked with the aid of Preview™ software, version 2.2.9 from Protein Metrics Inc. The effectiveness of tryptic digestions was verified before TMT labelling. Next, we checked for completeness of the labelling procedure. All checks were done as short 30-minute gradient with roughly 250 ng peptide injections. Missing cleavage was under 10% of all peptides and approx. 95% of all N termini and 100% of all lysines were successfully labelled with TMT tags. We identified 5,687 proteins in the icaRhoA series samples and 6,477 proteins in the dasatinib series samples. Among them there were 101 and 105 bovine proteins, respectively, coming from the fetal bovine serum used in the cell culture. For further analysis, we excluded these bovine proteins and all proteins with more than one missing intensity value in the sample series leading to 4,637 and 5,880 proteins, respectively usable for differential protein abundance analysis.

Principle component analysis of normalized gene expression and protein abundance profiles showed that the treatments, but also the biological replication are sources of significant variation (Fig. 4c). The paired character of the data thus has to be reflected in a linear model design formula in order to obtain the most accurate results from the statistical analyses of differential gene expression or differential protein abundance.

**Fig. 5:**
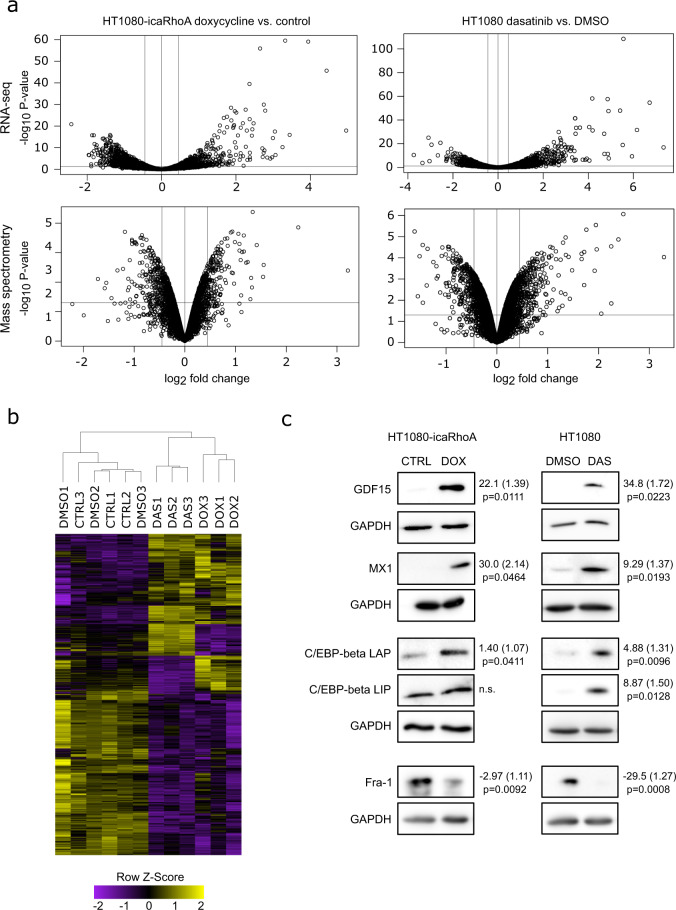
Analysis and validation of transcriptomic and proteomic results. (**a**) Volcano plots of transcripts and proteins affected by MAT in each treatment group. Vertical lines mark −1.5 and 1.5-fold change, respectively. Horizontal line marks statistical test P-value 0.05. (**b**) Unsupervised hierarchical clustering of genes that are differentially expressed after MAT in both treatment groups (P < 0.05). The yellow colour scale represents a higher expression level, and the purple colour scale represents a lower expression level. (**c**) Immunoblotting validation of differential protein abundance analysis results. GAPDH was used as a loading control in all experiments. Representative results of three independent experiments. Numbers next to blots indicate average fold change, standard error (both geometric) and p-value of paired t-test.

Differential gene expression analysis with DESeq2^38^ identified 894 out of 12,030 genes (7.4%) significantly changed (with adjusted P-value < 0.1) after induction of caRhoA. Dasatinib treatment changed the expression of 996 out of 12,637 (8.1%) genes. Differential protein abundance with limma R package^34^ revealed 410 out of 4,637 (8.8%) proteins significantly changed with adjusted P-value < 0.2 after induction of caRhoA. Dasatinib treatment changed the abundance of 674 out of 5,880 with adjusted P-value < 0.05 (11.5%) proteins. Note that we used a stricter adjusted P-value cut-off on the dasatinib data as the biological replicates were more uniform providing smaller P-values overall (with the opposite for the icaRhoA data). The differential gene and protein expression analysis results are summarized with volcano plots in Fig. 5a. Complete results are available from Figshare^34^ as “differential_expression_analyses.xlsx” spreadsheet file. The file also presents lists of genes and proteins concordantly affected by both MAT-inducing treatments in separate sheets. Joint hierarchical clustering of affected genes across both treatment sample series showed a high similarity in the overall impact of MAT on gene expression (Fig. 5b).

To validate the results of the high-throughput analyses, we performed immunoblotting experiments with independent biological samples (different from those used for the high-throughput analyses) to measure differences in protein abundance of four proteins – C/EBPβ, GDF15, MX1 and Fra-1 that were selected from those identified with both transcriptomic and proteomic profiling to be concordantly affected by both experimental treatments (see “Targets selected for verification” on Figshare^35^ for the detected fold changes and corresponding adjusted P-values). The blots confirmed reproducible differences in all four proteins in agreement with the data (Fig. 5c).

Taken together, we present a valuable transcriptomic and proteomic dataset characterizing fibrosarcoma cell response to induced migration mode switching. The dataset provides a useful resource for better understanding of the mechanisms of cancer metastatic spreading. The experimental model presented here has some limitations. Both treatments used in this work to induce MAT have their specific MAT-unrelated effects. The phenotypes induced by the treatments might be not identical. The mechanisms underlying the transition in HT1080 cells might be, at least in some details, different from those described in other cell types, particularly in melanoma cells. However, we believe that only continuing accumulation of data obtained from different cell contexts and with different experimental approaches will bring a better and more general understanding of the amoeboid migratory phenotype and its importance for cancer metastasis.

## Data Availability

The scripts used in data processing are available from Figshare^35^ (see “Code used in data processing”).

## References

[1] Hanahan D, Weinberg RA (2011). Hallmarks of cancer: the next generation. Cell.

[2] Vaškovičová K (2013). Invasive cells in animals and plants: searching for LECA machineries in later eukaryotic life. Biol. Direct.

[3] Tolde O (2018). Quantitative phase imaging unravels new insight into dynamics of mesenchymal and amoeboid cancer cell invasion. Sci. Rep..

[4] Parsons JT, Horwitz AR, Schwartz MA (2010). Cell adhesion: integrating cytoskeletal dynamics and cellular tension. Nat. Rev. Mol. Cell Biol..

[5] Tolde O, Rösel D, Veselý P, Folk P, Brábek J (2010). The structure of invadopodia in a complex 3D environment. Eur. J. Cell Biol..

[6] Nabeshima K, Inoue T, Shimao Y, Sameshima T (2002). Matrix metalloproteinases in tumor invasion: role for cell migration. Pathol. Int..

[7] Friedl P, Wolf K (2008). Tube Travel: The Role of Proteases in Individual and Collective Cancer Cell Invasion. Cancer Res..

[8] Wyckoff JB, Pinner SE, Gschmeissner S, Condeelis JS, Sahai E (2006). ROCK- and myosin-dependent matrix deformation enables protease-independent tumor-cell invasion *in vivo*. Curr. Biol..

[9] Wolf K (2003). Compensation mechanism in tumor cell migration: mesenchymal-amoeboid transition after blocking of pericellular proteolysis. J. Cell Biol..

[10] Lämmermann T (2008). Rapid leukocyte migration by integrin-independent flowing and squeezing. Nature.

[11] Charras GT (2008). A short history of blebbing. J. Microsc..

[12] Agarwal P, Zaidel-Bar R (2019). Diverse roles of non-muscle myosin II contractility in 3D cell migration. Essays in Biochemistry.

[13] Panková K, Rösel D, Novotný M, Brábek J (2010). The molecular mechanisms of transition between mesenchymal and amoeboid invasiveness in tumor cells. Cell. Mol. Life Sci..

[14] Pandya P, Orgaz JL, Sanz-Moreno V (2017). Modes of invasion during tumour dissemination. Mol. Oncol..

[15] Gandalovičová A (2017). Migrastatics—Anti-metastatic and Anti-invasion Drugs: Promises and Challenges. Trends in Cancer.

[16] Sanz-Moreno V (2011). ROCK and JAK1 Signaling Cooperate to Control Actomyosin Contractility in Tumor Cells and Stroma. Cancer Cell.

[17] Taddei ML (2014). Mesenchymal to amoeboid transition is associated with stem-like features of melanoma cells. Cell Commun. Signal..

[18] Čermák V (2018). RNA-seq of macrophages of amoeboid or mesenchymal migratory phenotype due to specific structure of environment. Sci. Data.

[19] Kosla J (2013). Metastasis of aggressive amoeboid sarcoma cells is dependent on Rho/ROCK/MLC signaling. Cell Commun. Signal..

[20] MacKay JL, Kumar S (2014). Simultaneous and independent tuning of RhoA and Rac1 activity with orthogonally inducible promoters. Integr. Biol..

[21] Ahn J, Sanz-Moreno V, Marshall CJ (2012). The metastasis gene NEDD9 product acts through integrin 3 and Src to promote mesenchymal motility and inhibit amoeboid motility. J. Cell Sci..

[22] Logue JS, Cartagena-Rivera AX, Chadwick RS (2018). c-Src activity is differentially required by cancer cell motility modes. Oncogene.

[23] Riedl J (2008). Lifeact: a versatile marker to visualize F-actin. Nat. Methods.

[24] Schindelin J (2012). Fiji: an open-source platform for biological-image analysis. Nat. Methods.

[25] Martin M (2011). Cutadapt removes adapter sequences from high-throughput sequencing reads. EMBnet.journal.

[26] Dobin A (2013). STAR: ultrafast universal RNA-seq aligner. Bioinformatics.

[27] Čermák V, Gandalovičová A, Merta L, Rösel D, Brábek J (2019). ArrayExpress.

[28] Erban T, Harant K, Chalupnikova J, Kocourek F, Stara J (2017). Beyond the survival and death of the deltamethrin-threatened pollen beetle Meligethes aeneus: An in-depth proteomic study employing a transcriptome database. J. Proteomics.

[29] Wang Y (2011). Reversed-phase chromatography with multiple fraction concatenation strategy for proteome profiling of human MCF10A cells. Proteomics.

[30] McAlister GC (2014). MultiNotch MS3 enables accurate, sensitive, and multiplexed detection of differential expression across cancer cell line proteomes. Anal. Chem..

[31] Vizcaíno JA (2016). 2016 update of the PRIDE database and its related tools. Nucleic Acids Res..

[32] Harant K, Čermák V, Rösel D, Brábek J (2020). PRIDE Archive.

[33] Gatto L, Lilley KS (2012). MSnbase-an R/Bioconductor package for isobaric tagged mass spectrometry data visualization, processing and quantitation. Bioinformatics.

[34] Ritchie ME (2015). limma powers differential expression analyses for RNA-sequencing and microarray studies. Nucleic Acids Res..

[35] Čermák V (2020). figshare.

[36] Koudelková L (2019). Novel FRET-Based Src Biosensor Reveals Mechanisms of Src Activation and Its Dynamics in Focal Adhesions. Cell Chem. Biol..

[37] Andrews, S. FastQC: a quality control tool for high throughput sequence data. [Online]. Available online at: http://www.bioinformatics.babraham.ac.uk/projects/fastqc/ (2010).

[38] Love MI, Huber W, Anders S (2014). Moderated estimation of fold change and dispersion for RNA-seq data with DESeq2. Genome Biol..

